# Design issues in crossover trials involving patients with Parkinson’s disease

**DOI:** 10.3389/fneur.2023.1197281

**Published:** 2023-08-21

**Authors:** David Sparrow, Deborah DeMolles, Ornella Dubaz, Raymon Durso, Bernard Rosner

**Affiliations:** ^1^VA Boston Healthcare System, Boston, MA, United States; ^2^Departments of Medicine and Public Health, Boston University Chobanian and Avedisian School of Medicine, Boston, MA, United States; ^3^Department of Neurology, Brigham and Women’s Hospital, Boston, MA, United States; ^4^Department of Biostatistics, Harvard T. H. Chan School of Public Health, Boston, MA, United States; ^5^Channing Division for Network Medicine, Brigham and Women’s Hospital, Boston, MA, United States; ^6^Harvard Medical School, Boston, MA, United States

**Keywords:** Parkinson’s disease, crossover study design, clinical trials, mixed models, carryover effect

## Abstract

**Background and objectives:**

Crossover designs are frequently used to assess treatments for patients with Parkinson’s disease. Typically, two-period two-treatment trials include a washout period between the 2 periods and assume that the washout period is sufficiently long to eliminate carryover effects. A complementary strategy might be to jointly model carryover and treatment effects, though this has rarely been done in Parkinson’s disease crossover studies. The primary objective of this research is to demonstrate a modeling approach that assesses treatment and carryover effects in one unified mixed model analysis and to examine how it performs in a simulation study and a real data analysis example, as compared to other data analytic approaches used in Parkinson’s disease crossover studies.

**Methods:**

We examined how three different methods of analysis (standard crossover *t*-test, mixed model with a carryover term included in model statement, and mixed model with no carryover term) performed in a simulation study and illustrated the methods in a real data example in Parkinson’s disease.

**Results:**

The simulation study based on the presence of a carryover effect indicated that mixed models with a carryover term and an unstructured correlation matrix provided unbiased estimates of treatment effect and appropriate type I error. The methods are illustrated in a real data example involving Parkinson’s disease. Our literature review revealed that a majority of crossover studies included a washout period but did not assess whether the washout was sufficiently long to eliminate the possibility of carryover.

**Discussion:**

We recommend using a mixed model with a carryover term and an unstructured correlation matrix to obtain unbiased estimates of treatment effect.

## 1. Introduction

In a crossover clinical trial, the effects of different treatments are administered on the same subject during different treatment periods (1, 2). A very common example of such a design is the two-period two-treatment design, often called the AB/BA design, where a subject is randomly assigned to either sequence AB or BA. A washout period may be included between the two periods to reduce carryover of the effect of treatment from one period to the next.

Crossover trials are being used more often and in a variety of clinical contexts. They are most appropriate for studies evaluating symptomatic treatment of diseases that are chronic or relatively stable (e.g., Parkinson’s disease [PD], rheumatoid arthritis), at least over the period under study (3). There continues to be debate on the analysis of treatment and carryover effects. We chose to examine methods of analysis being used in a defined clinical situation (PD) over the last 10 years.

The present paper considers several different methods to analyze crossover trials and examines their performance in a simulation study. The methods are illustrated in a PD crossover trial (4).

## 2. Methods

**Figure 1:**
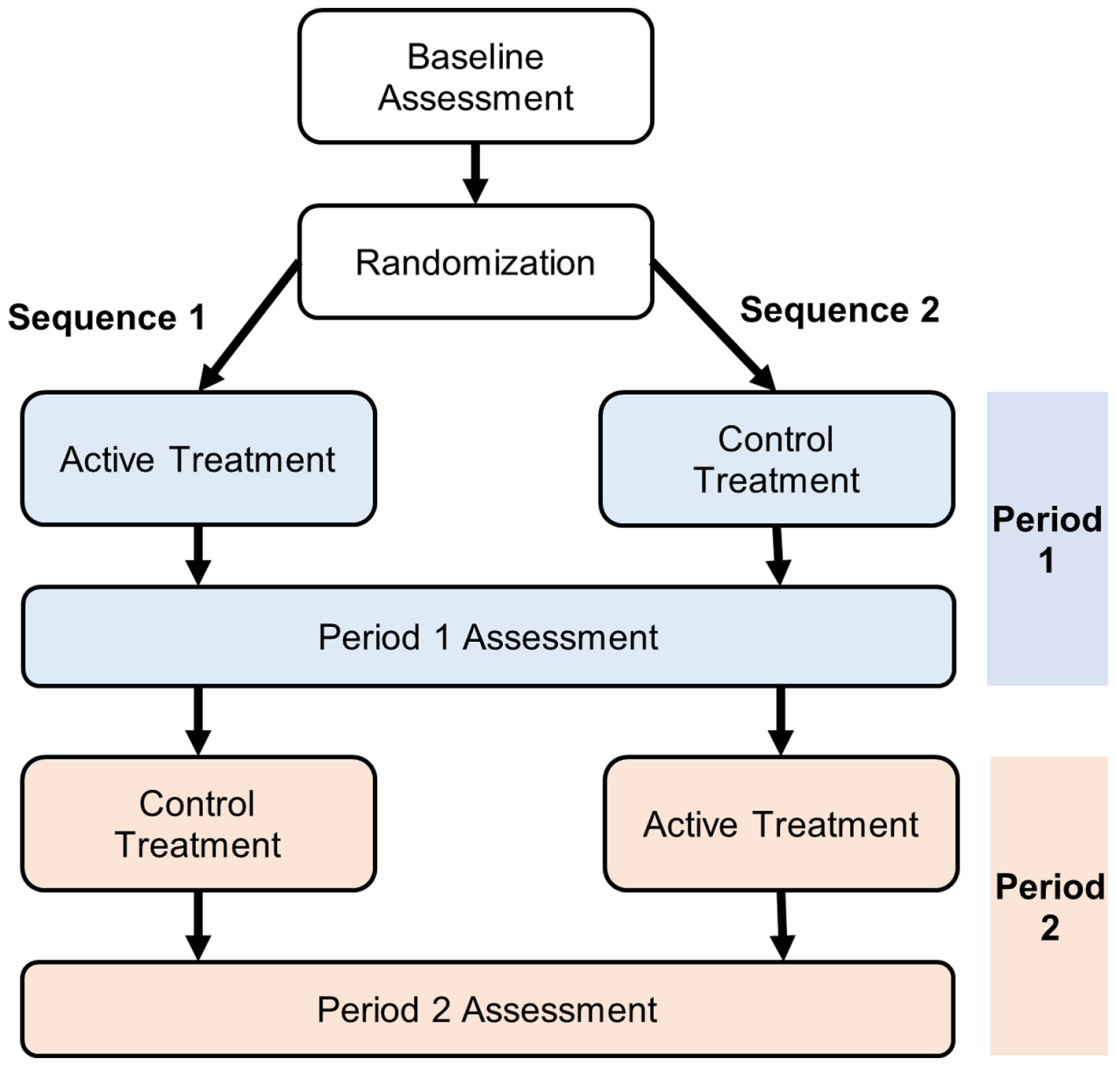
Two-period two-treatment crossover design.

We will describe approaches for the analysis of AB/BA crossover trials with an active treatment and a control treatment (i.e., no treatment/placebo) as illustrated in Figure 1. In this design, study participants undergo a baseline assessment and are then randomized to receive one of the two treatments (active or control) during period 1. At the end of the period 1, an assessment is conducted followed by a switch to the other treatment to be received during period 2 (with or without a washout period). At the end of period 2, a second assessment is conducted.

Some specific notation and definitions are used throughout this article. In general, let i = subject, j = assessment, where j = 0 if period 1 baseline, j = 1 if end of period 1, j = 2 if end of period 2. We will consider analytic strategies that assess effects of baseline, treatment, period, and carryover. Specifically, let:

y_ij_ = outcome value for the jth assessment of the ith subject (assumed continuous);

x_ij1_ = 1 if baseline, = 0 else, referred to subsequently as BASELINE;

x_ij2_ = 1 if active treatment (treatment 1), = 0 else, referred to subsequently as TREATMENT;

x_ij3_ = 1 if period 2, = 0 if period 1, referred to subsequently as PERIOD2;

x_ij4_ = 1 if carryover of treatment 1, = 0 else ≡ (1 − x_ij2_) x_ij3_, referred to subsequently as CARRYOVER;

S_i_ = sequence for the ith subject, = 1,2.

We assume that *n*_1_ subjects are randomly assigned to sequence 1 and n_2_ subjects to sequence 2, where *N* = *n*_1_ + *n*_2_ with 3 observations per subject.

*Sequence 1.* For sequence 1, subjects’ 1st period = active, 2nd period = control and 3 observations per subject are given by:

**Table:** 

Observation	j	x_ij1_	x_ij2_	x_ij3_	x_ij4_
1	0	1	0	0	0
2	1	0	1	0	0
3	2	0	0	1	1

*Sequence 2*. For sequence 2, subjects’ 1st period = control, 2nd period = active and 3 observations per subject are given by:

**Table:** 

Observation	j	x_ij1_	x_ij2_	x_ij3_	x_ij4_
1	0	1	0	0	0
2	1	0	0	0	0
3	2	0	1	1	0

The goal of the analysis is to estimate the treatment effect.

### 2.1. Methods of analysis

We consider three different methods of analysis that can be used for AB/BA crossover trials.

#### 2.1.1. Standard crossover *t*-test

For a standard crossover t-test, separate estimates of the mean of the within-subject differences between active and control are obtained for each sequence and separate variances of treatment effect are obtained in each sequence. The overall estimated treatment effect is an unweighted average of treatment effect estimates obtained in sequence 1 and 2 subjects and the variance is a weighted average of variance estimates obtained in sequence 1 and 2. Thus, the overall treatment effect is the unweighted average of the mean differences in outcome measurements between the active and control periods in sequence group 1 and sequence group 2, respectively. Specifically,


$$\mathrm{let}\;{\bar{d}}_{1}=\overset{n_{1}}{\underset{i=1}{\sum }}\left(y_{i1}-y_{i2}\right)/n_{1}\equiv \overset{n_{1}}{\underset{i=1}{\sum }}d_{i1}/n_{1},\mathrm{for subjects in sequence group}\;1$$

$${\bar{d}}_{2}=\overset{n_{2}}{\underset{i=1}{\sum }}\left(y_{i2}-y_{i1}\right)/n_{2}\equiv \overset{n_{2}}{\underset{i=1}{\sum }}d_{i2}/n_{2},\;\;\mathrm{for subjects in sequence group}\;2$$

$$\bar{d}=\left({\bar{d}}_{1}+{\bar{d}}_{2}\right)/2,$$

$$s_{d_{1}}^{2}=\overset{n_{1}}{\underset{i=1}{\sum }}{\left(d_{i1}-{\bar{d}}_{1}\right)}^{2}/\left(n_{1}-1\right),\mathrm{for subjects in sequence group}\;1$$

$$s_{d_{2}}^{2}=\overset{n_{2}}{\underset{i=1}{\sum }}{\left(d_{i2}-{\bar{d}}_{2}\right)}^{2}/\left(n_{2}-1\right),\mathrm{for subjects in sequence group}\;2$$

$$s_{d}^{2}=\left[\left(n_{1}-1\right)s_{d_{1}}^{2}+\left(n_{2}-1\right)s_{d_{2}}^{2}\right]/\left(n_{1}+n_{2}-2\right),$$

(1) $$t\_standard=\bar{d}/\sqrt{\left(s_{d}^{2}/4\right)\left(1/n_{1}+1/n_{2}\right)},\;$$

$$p-{\mathrm{value}}_{standard}=2\;x\;\mathrm{Pr}\left(t_{n_{1}+n_{2}-2}>\left|t\_standard\right|\right),$$

$$\mathrm{and}\;t_{n_{1}+n_{2}-2}\;\mathrm{is}\;a\;t\;\mathrm{distribution with}\;n_{1}+n_{2}-2\;\mathrm{degrees of freedom}.$$

This procedure assumes that there is no carryover effect of active treatment in period 1 on outcome in period 2.

#### 2.1.2. Mixed model with carryover term included in model statement

Because we have 3 repeated observations from each subject, which in general will be correlated with each other, we consider a mixed model approach to the analysis of the data. For this analysis, we use.

SAS PROC MIXED (Version 9.4, SAS Institute Inc., Cary, NC) with terms BASELINE (x_ij1_), TREATMENT (x_ij2_), PERIOD2 (x_ij3_), and CARRYOVER (x_ij4_) in the model statement. Specifically, we propose two different models:

a. Model 1: (mixed model with an unstructured covariance matrix)


(2) $$y_{ij}=\alpha +\overset{4}{\underset{k=1}{\sum }}\beta_{k}x_{ijk}+e_{ij},i=1,\ldots ,N;j=0,1,2\;$$

where


$$e_{i}=\left(e_{i0},e_{i1},e_{i2}\right)$$

$$e_{i}∼N\left(0,\Sigma_{i}\right),\mathrm{where}\;\Sigma_{i}=\sigma_{j_{1}}\sigma_{j_{2}}\rho_{j_{1}j_{2}};j_{1}=0,1,2;j_{2}=0,1,2$$

β_1_ = BASELINE effect, β_2_ = TREATMENT effect, β_3_ = PERIOD2 effect, and β_4_ = CARRYOVER effect, all mutually adjusted for each other.

In this setting, we allow for a different correlation between outcomes for each pair of assessments ( $\rho_{j_{1}j_{2}})$ and also a different variance at each assessment ( $\sigma_{j}^{2})$ . Thus, the correlation between the baseline observation and the end of the first period ( $\rho_{01}$ ) is assumed to be different from the correlation between the baseline and the end of the second period ( $\rho_{02}$ ) as well as the correlation between outcomes at the end of the first period vs. the end of the second period ( $\rho_{12}$ ).

We implemented this analysis by using the REPEATED statement with TYPE = UNR (with an unstructured covariance matrix, which allows for different variance estimates at each observation and unequal correlations between outcomes at different pairs of observations).

b. Model 2: (mixed model with a compound symmetry covariance matrix)


(3) $$y_{ij}=\alpha +a_{i}+\overset{4}{\underset{k=1}{\sum }}\beta_{k}x_{ijk}+e_{ij},i=1,\ldots ,N;j=0,1,2\;$$

where


$$a_{i}∼N\left(0,\sigma_{A}^{2}\right)\;\mathrm{and}\;e_{ij}∼N\left(0,\sigma^{2}\right)$$

In this setting, we assume that the within-subject variance ( $\sigma^{2}$ ) is the same at each time point, and the correlation ( $\rho =\sigma_{A}^{2}/(\sigma_{A}^{2}+$ $\sigma^{2}$ )) between outcomes for each pair of assessments are the same. This analysis was performed using the RANDOM statement (compound symmetry covariance matrix where the variances at each observation are forced to be the same and the correlation between outcomes at pairs of observations are also forced to be the same) with a random intercept.

The goal of the analysis is to estimate the TREATMENT effect (β_2_) and it’s variance (var(β_2_)) and to compare the variance when carryover is present (β_4_ ≠ 0) vs. when it is absent (β_4_ = 0), which we study in detail using simulation study analyses in Section 2.2.

#### 2.1.3. Mixed model with no carryover term

If we assume there is no carryover effect, we can use similar modeling approaches as in Section 2.1.2 but removing the term for CARRYOVER (x_ij4_). Therefore, equation 2 can be reduced to accommodate only BASELINE (x_ij1_), TREATMENT (x_ij2_), and PERIOD2 (x_ij3_) effects as follows:


(4) $$y_{ij}=\alpha +\overset{3}{\underset{k=1}{\sum }}\beta_{k}x_{ijk}+e_{ij},i=1,\ldots ,N;j=0,1,2\;$$

and, likewise, equation 3 can be reduced to the following:


(5) $$y_{ij}=\alpha +a_{i}+\overset{3}{\underset{k=1}{\sum }}\beta_{k}x_{ijk}+e_{ij},i=1,\ldots ,N;j=0,1,2\;$$

where the same notation is used as in equations 2 and 3.

The correlated data were accommodated using either the REPEATED or the RANDOM statement of SAS PROC MIXED in a similar manner as in Section 2.1.2 (model 1 or model 2, respectively).

### 2.2. Simulation study design

The simulation study will focus on the performance of the three methods of analysis listed above with simulation settings from the real data example using Mini-BESTest data (see Section 3.2 of Results below). Four thousand simulations were run for different parameter combinations. The *n* = 4,000 was chosen because one can show that in order to test that type I error is 0.05 with a one sample binomial test, one would have 80% power to reject the null hypothesis if the true type I error is 0.04. In each simulated sample, the sample size was set to 32, which is the approximate median sample size used in actual PD crossover trials (see Section 3.3 of Results), where 16 subjects were assigned to sequence 1 (active treatment followed by control) and 16 were assigned to sequence 2 (control followed by active treatment). Three outcome values were generated for each subject in the sample corresponding to outcomes obtained during (i) period 1 baseline, (ii) end of period 1, and (iii) end of period 2. The model used to simulate the outcome data, Y_it_ for the ith subject at time t, when a carryover effect was assumed to be present was:


(6) $$\begin{array}{c} Y_{it}=20.5+0.4\times \mathrm{BASELINE}+2\times \mathrm{TREATMENT}\\ -2\times \mathrm{PERIOD}2+1\times \mathrm{CARRYOVER}+e_{it}\; \end{array}$$

i.e. β_1_ = 0.4, β_2_ = 2, β_3_ = −2, and β_4_ = 1, and e_it_ is a random error term for the ith subject at time t. When no carryover effect was assumed, the term CARRYOVER was removed from equation 6 to simulate the data.

In the simulations, random errors were generated using SAS PROC SIMNORMAL with three different covariance structures as described in Sections 2.2.1 to 2.2.3.

#### 2.2.1. Compound symmetry

We set the pairwise correlations ρ_01_ = ρ_12_ = ρ_02_ = ρ for 3 different values of ρ (intraclass correlation coefficient [ICC]) = 0.8, 0.5, and 0.2, respectively. The covariance matrix was constructed with a common variance σ_0_^2^ = σ_1_^2^ = σ_2_^2^ = σ^2^ set to 13.4.

#### 2.2.2. Unstructured correlation, equal variance

We set ρ_01_ = 0.8, ρ_12_ = 0.2, and ρ_02_ = 0.1, where correlations are much stronger for outcomes within the same period (period 1 baseline and end of period 1) than for outcomes assessed at different periods (period 1 baseline and end of period 2; end of period 1 and end of period 2). The covariance matrix was constructed with a common variance σ_0_^2^ = σ_1_^2^ = σ_2_^2^ = σ^2^ set to 13.4.

#### 2.2.3. Unstructured correlation, unequal variance

We set ρ_01_ = 0.8, ρ_12_ = 0.2, and ρ_02_ = 0.1, but allowed the variances to be heterogeneous as well (σ_0_^2^ = 17, σ_1_^2^ = σ_2_^2^ = 11).

Several performance measures, including mean and variance of estimated treatment effect, bias, coverage, power, and type I error were obtained from the simulations.

### 2.3. Crossover trials with 4 observations

The mixed model defined in equation 2 can be extended to allow for 4 observations with baseline measurements at the beginning of each of two periods. The design matrices for this situation are given below.

*Sequence 1*. For sequence 1, subjects’ 1st period = active, 2nd period = control and 4 observations per subject are given by:

**Table:** 

Observation	j	x_ij1_	x_ij2_	x_ij3_	x_ij4_
1	0	1	0	0	0
2	1	0	1	0	0
3	2	1	0	1	1
4	3	0	0	1	1

*Sequence 2*. For sequence 2, subjects’ 1st period = control, 2nd period = active and 4 observations per subject are given by:

**Table:** 

Observation	j	x_ij1_	x_ij2_	x_ij3_	x_ij4_
1	0	1	0	0	0
2	1	0	0	0	0
3	2	1	0	1	0
4	3	0	1	1	0

The mixed model based on 4 observations is given in equation 7.


(7) $$y_{ij}=\alpha +\overset{4}{\underset{k=1}{\sum }}\beta_{k}x_{ijk}+e_{ij},i=1,\ldots ,N;j=0,1,2,3\;.$$

where the $x_{ijk}\;$ are defined similarly to the 3-observation design in equation 2.

### 2.4. Systematic review

Crossover trials are being used more often and in a variety of clinical contexts, including PD. We searched PubMed, EMBASE, CINAHL, and Health & Medical Collection between 2012 and 2021 with the combination of the search terms “Parkinson disease” and “crossover.” The search strategy was adapted for multiple databases. A detailed description of the search procedure is provided in Supplementary Table S1, which may be found in the online version of this article at the publisher’s web-site.

## 3. Results

### 3.1. Simulation results

For designs with three observations, we considered several different methods for simulating the data presented in Panels 1–6 and for each Panel several different methods for analyzing the data (see Table 1).

**Table 1:** Results according to simulation design and method of analysis.

Panel	Simulation design		Analysis			Mean of estimated treatment effect	Variance of estimated treatment effect	Bias^a^	Coverage^b^	Power^c^	Type I error^d^
	Structure	Carryover	Method	Carryover term	SAS procedure						
1	CS^e^	Yes^f^	T-TEST		T-TEST	1.5	0.42	−0.5	88.2%	61.0%	5.1%
			MIXED	No^g^	RANDOM^i^	1.5	0.42	−0.5	88.2%	62.8%	5.1%
			MIXED	Yes^h^	RANDOM^i^	2.0	1.27	0.0	95.2%	42.6%	4.8%
2	CS^e^	No^j^	T-TEST		T-TEST	2.0	0.42	0.0	94.9%	83.7%	5.1%
			MIXED	No^g^	RANDOM^i^	2.0	0.42	0.0	94.9%	85.4%	5.1%
			MIXED	Yes^h^	RANDOM^i^	2.0	1.27	0.0	95.2%	42.6%	4.8%
3	UNR^k^	Yes^f^	T-TEST		T-TEST	1.5	0.67	−0.5	90.9%	43.4%	5.2%
			MIXED	No^g^	RANDOM^i^	1.5	0.67	−0.5	86.4%	52.8%	8.1%
			MIXED	Yes^h^	RANDOM^i^	2.0	0.92	0.0	98.6%	34.0%	1.5%
4	UNR^k^	Yes^f^	T-TEST		T-TEST	1.5	0.67	−0.5	90.9%	43.4%	5.2%
			MIXED	No^g^	REPEATED^l^	1.7	0.39	−0.3	92.3%	75.2%	5.0%
			MIXED	Yes^h^	REPEATED^l^	2.0	0.63	0.0	95.4%	67.6%	4.6%
5	UNR^m^	Yes^f^	T-TEST		T-TEST	1.5	0.55	−0.5	90.3%	50.4%	5.2%
			MIXED	No^g^	REPEATED^l^	1.7	0.32	−0.3	91.8%	81.5%	5.0%
			MIXED	Yes^h^	REPEATED^l^	2.0	0.52	0.0	95.4%	76.1%	4.6%
6	CS^e^	Yes^f^	MIXED	No^g^	REPEATED^l^	1.5	0.45	−0.5	88.8%	58.4%	5.0%
			MIXED	Yes^h^	REPEATED^l^	2.0	1.30	0.0	95.4%	40.1%	4.7%

In all simulation designs, we assume a sample size of 32 subjects and a treatment effect of 2.0 (4,000 datasets per simulation design).

a Bias (mean estimated treatment effect - true treatment effect).

b Coverage (% simulated samples where true treatment effect lies within the 95% confidence interval of estimated treatment effect).

c Power (% simulated samples where estimated treatment effect was significant at α = 0.05).

d Type I error determined under the assumption of no treatment effect (null hypothesis), calculated as the % of samples in which the estimated treatment effect was significant at α = 0.05.

e Compound symmetry covariance matrix used to generate random errors with variance of 13.4 and ICC of 0.5.

f Carryover effect set to 1.0.

g Mixed model with no carryover term in model statement.

h Mixed model with carryover term included in model statement.

i Mixed models run with RANDOM statement (compound symmetry correlation structure).

j Carryover effect set to 0.

k Unstructured correlation matrix was used to generate random errors with variance of 13.4 and pairwise correlations ρ_01_ = 0.8, ρ_12_ = 0.2, and ρ_02_ = 0.1.

l Mixed models run with REPEATED statement and TYPE = UNR (unstructured correlation structure).

m Unstructured correlation matrix was used to generate random errors with unequal variances σ_0_^2^ = 17, σ_1_^2^ = σ_2_^2^ = 11 and pairwise correlations ρ_01_ = 0.8, ρ_12_ = 0.2, and ρ_02_ = 0.1.

#### 3.1.1. Table 1, Panel 1

The three methods of analysis were performed on the first set of simulated data based on a compound symmetry correlation structure with an ICC of 0.5 and a carryover effect of 1. The standard crossover t-test provides a biased estimate of treatment effect and has coverage lower than the nominal 95% coverage. The type I error is appropriate. The results of the mixed model with no carryover term (equation 4) are virtually identical to those of the crossover t-test. The mixed model that includes a carryover term provides an unbiased treatment estimate, appropriate coverage probability, and appropriate type I error. In general, differences between the mixed model with a carryover term and the other two methods are greatest when the correlations between repeated measures is high. Similar conclusions are obtained for the other ICC values (0.2, 0.8; results not shown); hereafter, for ease of presentation, only the results for ICC = 0.5 are presented.

#### 3.1.2. Table 1, Panel 2

With the carryover effect set to 0, the standard crossover t-test and the mixed model with no carryover term have no bias, appropriate coverage, and appropriate type I error. The mixed model with a term for carryover also has no bias, correct coverage, and correct type I error, but does suffer in terms of power compared to the mixed model with no carryover term and the standard crossover *t*-test.

#### 3.1.3. Table 1, Panel 3

For the simulated data based on a more general unstructured (heterogeneous) correlation structure and a carryover effect, the standard crossover t-test has a correct type I error; however, the treatment estimate is biased and the coverage is too low. The mixed model with no carryover term and a covariance matrix constructed with common variance has an incorrect type I error, is biased, and has coverage probability too low. The mixed model with a carryover term has no bias but the type I error is too low and the coverage is too high. These mixed models were run using the RANDOM statement for repeated measures, which assumes a compound symmetry correlation structure, which differs from the actual correlation structure of the data (unstructured correlation structure). The results suggest the need for a modeling approach that assumes a different correlation structure.

#### 3.1.4. Table 1, Panels 4 and 5

We repeated the mixed models (with and without a carryover term) with the use of the REPEATED statement with TYPE = UNR (unstructured correlation structure; Panel 4). This provided an appropriate type I error with the carryover term included. However, the model with no carryover term still has low coverage and is biased, whereas the model with a carryover term (equation 2) is unbiased with appropriate coverage. We repeated the simulation described in Panel 4 but generated the data so that the standard deviations were different for the three observations (σ_0_^2^ = 17, σ_1_^2^ = σ_2_^2^ = 11). The modeling (with and without a carryover term) results (Panel 5) were comparable to those in Panel 4 in terms of bias, coverage, and type I error. Power for the mixed model with carryover term increased from 68 to 76%. This is probably because the unstructured correlation structure used to analyze the data allows for heterogeneous variances of the three repeated measures, which is more consistent with the simulation design in Panel 5 where the variances were allowed to be different than the simulation design in Panel 4 where they were forced to be the same.

#### 3.1.5. Table 1, Panel 6

We repeated the mixed models in Table 1, Panel 1 replacing the RANDOM statement with a REPEATED statement with an unstructured correlation matrix. With the use of the REPEATED statement, there is still an appropriate type I error but a slight reduction in power (43% vs. 40% if carryover term is present and 63% vs. 58% if carryover term is absent). Also, the model with a carryover term present continues to provide an unbiased estimate of treatment effect and appropriate 95% coverage. The fact that modeling with use of the REPEATED statement with an unstructured correlation matrix can accommodate many different types of correlation structure that may be present in the data argues for using this method.

### 3.2. Real data example

Data from a real life two-period two-treatment crossover trial is used to illustrate our methods of analysis. The study evaluated the efficacy of 3 months of balance exercise training compared with usual care on the outcomes of dynamic balance (Mini-BESTest) and fear of falling (Falls Efficacy Scale-International [FES-I]) in patients with PD (4).

#### 3.2.1. Mini-BESTest

Subjects in both sequences improved in dynamic balance (as assessed by Mini-BESTest) with exercise vs. usual care; however, there was more improvement in sequence 2 suggesting the possibility of a carryover effect (Table 2, Section A). We conducted a mixed model analysis with a carryover term and observed a significant treatment effect and a significant positive carryover effect (Table 2, Section Ba). Table 2, Section Bb indicates that there is greater variance at baseline than in periods 1 and 2 and that the correlation structure is mildly divergent from compound symmetry. The estimated treatment effect from the standard crossover t-test, which does not adjust for carryover, is substantially smaller than that of the mixed model with a carryover term (Table 2, Section C). Our results from Table 1, Panel 4 suggest that it is smaller because it is biased and, therefore, an underestimate of the treatment effect.

**Table 2:** Analysis of a balance exercise training intervention for PD patients: descriptive statistics and alternative methods of analysis for the Mini-BESTest.^a^

A. Descriptive statistics						
	Sequence 1 (Exercise ➔ Usual care) *N* = 7			Sequence 2 (Usual care ➔ Exercise) *N* = 9		
	Treatment	Mean	Standard error	Treatment	Mean	Standard error
Baseline	None	21.4	1.76	None	20.6	1.29
Period 1	Exercise	25.0	0.87	Usual care	20.2	1.24
Period 2	Usual care	24.1	0.80	Exercise	22.3	1.51
Exercise minus usual care		0.9	0.63		2.1	1.02

a Higher values of Mini-BESTest correspond to better balance.

b Mixed models with the use of SAS PROC MIXED, REPEATED option, TYPE = UNR (unstructured correlation matrix).

c The diagonal elements contain the variances at specific time points; the off diagonal elements contain the estimated Pearson correlations between outcomes at different time periods.

#### 3.2.2. FES-I

Subjects in both sequences had less fear of falling (as assessed by FES-I) with exercise vs. usual care; however, there was less fear in sequence 2 again suggesting the possibility of a carryover effect (Table 3, Section A). We conducted a mixed model analysis with a carryover term and observed no significant carryover effect (Table 3, Section Ba). There is strong evidence of heterogenous variance over different periods with a higher variance for baseline measurements than during subsequent periods (Table 3, Section Bb). In addition, there is also strong evidence of heterogeneous correlation with higher within period 1 correlation (0.86) and lower between period 1 and 2 correlation (0.41 to 0.46). The estimated treatment effect from the standard crossover t-test is slightly smaller than that of the mixed model with a carryover term (Table 3, Section C).

**Table 3:** Analysis of a balance exercise training intervention for PD patients: descriptive statistics and alternative methods of analysis for the FES-I^a^.

**A. Descriptive statistics**						
	Sequence 1 (Exercise ➔ Usual care) *N* = 7			Sequence 2 (Usual care ➔ Exercise) *N* = 9		
	Treatment	Mean	Standard error	Treatment	Mean	Standard error
Baseline	None	29.3	2.87	None	27.6	2.47
Period 1	Exercise	24.1	2.69	Usual care	27.1	1.76
Period 2	Usual care	26.0	2.88	Exercise	22.7	1.15
Exercise minus usual care		−1.9	2.32		−4.4	2.01

a Lower values of FES-I correspond to less fear of falling.

b Mixed models with the use of SAS PROC MIXED, REPEATED option, TYPE = UNR (unstructured correlation matrix).

c The diagonal elements contain the variances at specific time points; the off diagonal elements contain the estimated Pearson correlations between outcomes at different time periods.

### 3.3. Results of the literature search

Our initial search retrieved 691 abstracts of potential interest. After full text review and consideration of inclusion criteria (Supplementary Table S1), 36 AB/BA crossover trials in PD with an active treatment and a control treatment were identified and evaluated (Table 4). There was no evidence that any of these studies controlled for carryover. Furthermore, only six provided some information to suggest that carryover was minimal: in two studies, the washout period was seemingly long enough based on pharmacokinetics (25, 33); in four other studies, a test for carryover was performed but not statistically significant (8, 9, 13, 17). However it’s well known that tests for carryover often have low statistical power (40).

**Table 4:** Characteristics of included studies.

Study	Participants		Washout period	Evaluated carryover (Yes/No)	Method of analysis
	Intervention	Control			
Di Giacopo et al. (5)	Rivastigmine patch; 4.6 mg/day for 3 weeks	Placebo patch	7 days	No	Friedman test
Gelfin et al. (6)	D-serine; 30 mg/kg/day for 6 weeks	Placebo	3 weeks	No	Repeated measures ANOVA
Randhawa et al. (7)	rTMS; 5 Hz applied over SMA for 6 min one time	Sham simulation	1 week	No	Two-way ANOVA
Arii et al. (8)	Repetitive trans-spinal magnetic stimulation; 5 Hz applied over maximal anteflexion part of spinal column one time (40 total stimuli)	Sham stimulation	1 week	Yes	Ordinary paired *t-*test*
Fietzek et al. (9)	Exercise with cueing; 2 times a week for 30 min over 2 weeks	Usual care	0	Yes	Standard crossover *t*-test
Hattori et al. (10)	Apomorphine hydrochloride; one-time administration	Placebo	1 day	No	Linear mixed model
Neikrug et al. (11)	CPAP; 3 weeks	Placebo	0	No	Ordinary paired *t-*test*
Valentino et al. (12)	tDCS; applied over primary motor cortex (M1) for 5 days	Sham stimulation	3 months	No	Two-way ANOVA
Bernard et al. (13)	Sildenafil over 4 weeks	Placebo	4 weeks	Yes	Ordinary unpaired two-sample *t-*test
Flamez et al. (14)	Single session study: rTMS; 1 Hz applied over primary motor cortex for 16 min once	Sham stimulation	1 week	No	Two-way repeated measures ANOVA
	Multi session study: rTMS; 1 Hz applied over primary motor cortex for 32 min twice per day over 5 days	Sham stimulation	4 weeks	No	Two-way repeated measures ANOVA
Sparrow et al. (4)	Balance exercise; twice weekly for 90 min over 3 months	Usual care	0	Yes	Standard crossover *t*-test
Fox et al. (15)	Dextromethorphan/quinidine (45 mg/10 mg); twice daily over 2 weeks	Placebo	2 weeks	No	Repeated measures ANOVA
Bruno et al. (16)	Botulinum toxin type A; multiple injections in upper and lower limbs one time	Placebo	12 weeks	No	Wilcoxon signed-rank test
Büchele et al. (17)	Sodium Oxybate; drinkable solutions 2 times per day for 6 weeks	Placebo	2–4 weeks	Yes	Linear mixed model
Cheng et al. (18)	Dihydroergotoxin mesylate; 2.5 mg twice daily for 2 weeks	Placebo	1 week	No	Wilcoxon signed-rank test
Corvol et al. (19)	Naftazone; 160 mg/day for 14 days	Placebo	1–2 weeks	No	Linear mixed model
Fricke et al. (20)	rTMS; 1 Hz applied over primary motor cortex and dorsal premotor cortex one time	Sham stimulation	~1 week	No	Repeated measures ANOVA
Gourcerol et al. (21)	STN-DBS; stimulator turned ON for 2 h one time	Stimulator turned OFF	0	No	Wilcoxon signed-rank test
Hauser et al. (22)	Orally inhaled levodopa (CVT-301); inhaler provided dose of 84 mg one time	Placebo	1–7 days	No	Extension of the Wilcoxon-Gehan rank-sum test for crossover data
Peppe et al. (23)	Proprioceptive focal stimulation using Equistasi ®; Equistasi device worn 8 weeks	Sham stimulation	4 weeks	Yes	Repeated measures ANOVA
de Faria et al. (24)	Cannabidiol; 300 mg one time	Placebo	15 days	No	Repeated measures ANOVA
Delgado-Lara et al. (25)	Melatonin; 25 mg twice daily for 3 months	Placebo	4 days	Yes	Mann–Whitney U test
Fabbri et al. (26)	STN-DBS; stimulator turned ON for 60 min one time	Stimulator turned OFF	0	No	Wilcoxon signed-rank test
Hasegawa et al. (27)	Agility Boot Camp with Cognitive Challenges; group exercise class 3 times a week for 6 weeks	Group education classes	0	No	Linear mixed model
Jung et al. (28)	Agility Boot Camp with Cognitive Challenges; group exercise class 3 times a week for 6 weeks	Group education classes	0	No	Linear mixed model
King et al. (29)	Agility Boot Camp with Cognitive Challenges; group exercise class 3 times a week for 6 weeks	Group education classes	0	No	Linear mixed model
Lohse et al. (30)	rTMS; 1 Hz applied over preSMA for 30 min one time	Sham stimulation	2 weeks	No	Wilcoxon signed-rank test
Meloni et al. (31)	5-HTP; 50 mg daily over 4 weeks	Placebo	4 weeks	No	Two-way ANOVA
Meloni et al. (32)	5-HTP; 50 mg daily over 4 weeks	Placebo	4 weeks	No	Two-way ANOVA
Stuart et al. (33)	Donepezil; 5 mg per day for 2 weeks	Placebo	2 weeks	No	Linear mixed model
Yuan et al. (34)	Video game-based exercise; 30 min, 3 days a week for 6 weeks	Usual care	0	No	Repeated measures ANOVA within sequence groups
Albin et al. (35)	Varenicline; 0.5 mg b.i.d. for 3 weeks	Placebo	3 weeks	No	Linear mixed model
Brugger et al. (36)	Intermittent theta burst stimulation; applied over supplementary motor cortex one time	Sham stimulation	1 to 4 weeks	No	Linear mixed model
Mishra et al. (37)	tDCS; constant electric current of 2 mA delivered over left dorsolateral prefrontal cortex for 30 min one time	Sham stimulation	≥1 week	No	Two-way repeated measures ANOVA
Plastino et al. (38)	Safinamide; 50 mg a day for 3 months	Usual care	15 days	No	Ordinary paired *t*-test *
Vitorio et al. (39)	Donepezil; 5 mg a day for 2 weeks	Placebo	2 weeks	No	Linear mixed model

ANOVA, analysis of variance; 5-HTP, 5-hydroxytryptophan; mA, milliamperes; rTMS, repetitive transcranial magnetic stimulation; SMA, supplementary motor area; STN-DBS, subthalamic deep brain stimulation; tDCS, transcranial direct current stimulation.

* The estimate of the treatment effect is obtained as the overall (not considering sequence group) mean of the within-subject differences between active and control.

Eighteen of the 36 AB/BA crossover trials evaluated a drug intervention (5, 6, 10, 13, 15–19, 22, 24, 25, 31–33, 35, 38, 39); all 18 included a washout period (range, 1 day to 12 weeks). Washout period duration can be based on estimates of drug half-life and the number of days to reach a steady state as in two of these trials (25, 33). The remaining 16 studies did not provide a justification for the washout period duration. Of these 16, only two investigated carryover effect and both of these studies found it nonsignificant; however, it is not clear how these investigations were done (13, 17). Despite these nonsignificant findings, a residual carryover effect may not be negligible and thus should be controlled (41, 42).

Eighteen of the 36 AB/BA crossover trials evaluated an intervention other than a drug (exercise, transcranial direct current stimulation [tDCS], repetitive transcranial magnetic stimulation [rTMS], proprioceptive focal stimulation, subthalamic deep brain stimulation [STN-DBS], theta burst stimulation, CPAP) (4, 7–9, 11, 12, 14, 20, 21, 23, 26–30, 34, 36, 37). Nine of these trials did not include a washout period and provided no justification whereas nine included a washout period but provided no justification for the length of the washout period.

## 4. Discussion

AB/BA crossover studies can produce results that are statistically and clinically valid, and the sample size required for a given power is typically much smaller, compared to that of a parallel-group design. The smaller sample size required in crossover studies is an important advantage because patient recruitment is often a problem in clinical research (43–45), particularly in PD. Evidence suggests that insufficient enrollment prevents completion of 30% of PD clinical trials and delays 85% of those eventually completed (46). In addition, research that is underpowered (due to small sample size) will have a higher probability of failing to detect true therapeutic benefits, potentially resulting in missed opportunities to utilize effective interventions (47). Furthermore, in our experience with treatment trials using a placebo as a comparator and a parallel group design, many subjects are put off by the chance that they may not receive the active treatment. In a crossover trial all patients will receive the active treatment, which helps many subjects to legitimize the time and effort that goes into being a study participant and enhances recruitment possibilities.

A problem particularly associated with crossover trials is a residual carryover effect (e.g., a drug carryover effect when traces of the period 1 drug persist). This is a particular concern in trials with no washout period, which in our review occurred in 25% of the trials. Although many crossover trials include a washout period as a means of reducing carryover, we found that a substantial number of trials lacked justification for the length of the washout period, which is also very concerning. Even when a washout of an active treatment is completely effective, physiological or psychological states induced by the treatment may linger into the subsequent period (41, 48). But the required length of a physiological/psychological washout period is usually unknown.

Some authors have argued that the use of the crossover design is effectively built on the assumption that there is minimal carryover of the effect of a treatment into the next period (49, 50). Following this philosophy, rather than addressing carryover, one should proceed as if there were no carryover. Therefore, it is not surprising that our literature review revealed no evidence that any of the 36 AB/BA crossover trials controlled for carryover effect, which is particularly problematic for studies without a washout period (4, 9, 11, 21, 26–29, 34). Fortunately, researchers are increasingly including a washout period.

This study aims at assessing different strategies for dealing with potential carryover in crossover trials. Crossover trials should be designed with a sufficiently long washout period. Prior trials can be used to provide information on designing an adequate washout period (1, 2). Additionally, for drug intervention studies, washout period duration can be based on estimates of drug half-life and the number of days to reach a steady state. On the other hand, having too long of a washout period could lead to significant loss of subjects. In addition, including a longer washout period could alter results due to disease progression or other lifestyle circumstances (i.e., unrelated illness, fatigue, change in social circumstances). In addition to carefully choosing a washout period, a complementary analytical strategy, which we advocate, is to jointly model carryover, period, and treatment effects in the interest of obtaining unbiased estimates of treatment effect, whether carryover effect is statistically significant or not.

With the use of a mixed model with carryover term we avoided such bias in each set of simulated data. Such modeling performed with the use of an unstructured correlation structure (equation 2) resulted in the most appropriate type I error and coverage probability. In contrast, modeling with the use of an incorrect correlation structure, specifically compound symmetry (Table 1, Panel 3), and including a carryover term, has a large effect on the type I error (i.e., an invalid test procedure), whether a true carryover effect is present or not. Conversely, if an unstructured correlation structure is used for modeling and compound symmetry was the true correlation structure, type I error and coverage probability are preserved and power is only slightly reduced.

Thus, it appears the most conservative approach is to use a mixed model with a carryover term and an unstructured correlation structure (equation 2). With this approach we obtain unbiased estimates of treatment effect both with and without carryover effect present and obtain appropriate coverage regardless of the actual correlation structure in the data. Therefore, we recommend that researchers apply this modeling approach in the analysis of all AB/BA crossover trials with an active treatment and a control treatment and a single baseline.

Although we have concentrated on bias arising from carryover effect in this paper, we would be remiss not to mention other sources of bias: bias arising from period effect (1, 51) or missing outcome data (2, 51). Bias due to period effect may occur when the disease changes systematically over time, or if there are changes over time in background factors such as underlying medical management strategies. However, potential bias arising from period effect can be overcome by using a statistical analysis that includes period effect, such as in our mixed model analyses featured in this article. Bias due to missing outcome data may occur because a participant drops out in the second treatment period because of a poorer experience than in the first period.

In this paper, we provide modeling strategies for crossover studies with either 3-observation or 4-observation designs and provide simulation results for alternative modeling strategies in the 3-observation setting. It is likely that unbiased estimates of treatment effect can be obtained with both 3-observation and 4-observation designs, but it would be expected that the standard errors of parameter estimates would be somewhat smaller with the 4-observation design. On the other hand, dropout is more likely with a 4-observation vs. a 3-observation design since patient burden is increased. A detailed simulation study comparing the efficiency of these 2 designs will be the subject of another paper.

In this paper, we have discussed several modeling options for crossover design and present detailed SAS code for implementing these modeling options (Supplementary Datasheet S1). Mixed model analyses are also available in Stata (using the mixed command), R (using the lmer command), and SPSS (using the mixed command), although specific options available may vary across different packages.

In summary, mixed model analyses offer the opportunity to realize the advantages of crossover designs in obtaining unbiased estimates of treatment effect by simultaneously modeling treatment, period, and carryover effects and providing for an appropriate correlation structure among the repeated measures. We recommend this analytic strategy to fully take advantage of the reduced sample size needed for crossover designs while still obtaining unbiased estimates of treatment effects.

## Data availability statement

The raw data supporting the conclusions of this article will be made available by the authors, without undue reservation.

## Ethics statement

Ethical review and approval was not required for the study on human participants in accordance with the local legislation and institutional requirements. Written informed consent for participation was not required for this study in accordance with the national legislation and the institutional requirements.

## Author contributions

DS: research project: conception, execution, statistical analysis: review and critique, manuscript preparation: writing of the first draft, and review and critique. DD and BR: research project: organization, execution, statistical analysis: design, execution, review and critique, manuscript preparation: writing of the first draft, and review and critique. OD and RD: research project: organization, statistical analysis: review and critique, manuscript preparation: review and critique. All authors contributed to the article and approved the submitted version.

## Funding

The work in this manuscript was supported by VA Rehabilitation Research and Development.

## Conflict of interest

The authors declare that the research was conducted in the absence of any commercial or financial relationships that could be construed as a potential conflict of interest.

## Publisher’s note

All claims expressed in this article are solely those of the authors and do not necessarily represent those of their affiliated organizations, or those of the publisher, the editors and the reviewers. Any product that may be evaluated in this article, or claim that may be made by its manufacturer, is not guaranteed or endorsed by the publisher.
