# Supplementary material for: Design issues in crossover trials involving patients with Parkinson’s disease
Source: Front Neurol. 2023 Aug 21;14:1197281. doi: 10.3389/fneur.2023.1197281 (PMC10476358; doi:10.3389/fneur.2023.1197281)
Supplement: Supplementary file 1 [file Table_1.DOCX]

Literature Search Strategy

Our goal was to identify AB/BA crossover trials in PD with an active treatment and a control treatment. We searched PubMed, EMBASE, CINAHL, and Health & Medical Collection databases with the combination of the search terms “Parkinson disease” and “crossover”. Only articles in English published between 2012 and 2021 were considered. The exact search strategy for each database searched are shown in Table S1. All searches were completed on June 2, 2022. In addition, we reviewed the reference lists of each article reviewed for additional crossover studies to consider. In total, we identified 691 unique articles (616 results from PubMed; 348 results from EMBASE of which 51 were unique; 138 results from CINAHL of which 4 were unique; 111 results from Health & Medical Collection of which 20 were unique). Duplicate articles were eliminated with the use of the EndNote reference management application.

| Table S1. Search strategies for specific databases | | | |
| --- | --- | --- | --- |
| Database |  | Specific search terms used |  |
| PubMed |  | ((parkinson's OR "Parkinson Disease"[Mesh]) AND (crossover OR cross-over)) NOT (cross-sectional)  Filters: English, from 2012 – 2021 |  |
| EMBASE |  | ('parkinson disease'/exp OR parkinson) AND (crossover OR 'cross over') AND ([article]/lim OR [article in press]/lim OR [conference paper]/lim OR [conference review]/lim OR [review]/lim) AND [english]/lim AND [2012-2021]/py NOT 'cross sectional' |  |
| CINAHL |  | ((MH "Parkinson Disease") OR parkinson's) AND (crossover OR cross-over)) NOT cross-sectional  Limiters - Published Date: 20120101-20211231; English Language; Peer Reviewed |  |
| Health & Medical Collection |  | (mesh.Exact("Parkinson Disease") OR noft(parkinson's)) AND noft((crossover OR cross-over) NOT cross-sectional)  Limits applied  Limited by: Peer reviewed  Date: From January 01 2012 to December 31 2021  Document type: Article  Language: English |  |

The titles and abstracts of articles of potential interest were studied by two reviewers (D.S. and D.D.) independently, and selected studies underwent full-text review. Any disagreement raised between the reviewers was resolved through discussion and referral to a third reviewer (B.R.) where necessary, till mutual consensus amongst all.
