# Supplementary material for: Design issues in crossover trials involving patients with Parkinson’s disease
Source: Front Neurol. 2023 Aug 21;14:1197281. doi: 10.3389/fneur.2023.1197281 (PMC10476358; doi:10.3389/fneur.2023.1197281)
Supplement: Supplementary file 2 [file Data_Sheet_1.DOCX]

/**--

THIS CODE ASSUMES DATASET “ALL” HAS 3 SEPARATE OBSERVATIONS:

AT BASELINE, AT END OF PERIOD #1, AT END OF PERIOD #2

---**/

*****************************************************************;

*** CROSS OVER TTEST (DBAR) ***;

**--CREATE DATASET FOR CROSS OVER TTEST--**;

data crossover1;

** need to create one obs per subj with t1 & t2 (remove baseline) **;

do until (last.patientID);

set all; by patientID;

where BASELINE=0; **--remove baseline, just period 1 & period 2--**;

** first period **;

if PERIOD2=0 then do; OUTCOME1=OUTCOME; ACTIVE1=ACTIVE; end;

** second period **;

else if PERIOD2=1 then do; OUTCOME2=OUTCOME; ACTIVE2=ACTIVE; end;

end;

** calculate difference = active value - control value **;

** when period 1 is active, diff = t1 – t2 **;

if ACTIVE1=1 then diff_OUTCOME = OUTCOME1-OUTCOME2;

** otherwise, diff = t2 – t1 **;

else diff_OUTCOME = OUTCOME2-OUTCOME1;

keep patientID OUTCOME1 OUTCOME2 ACTIVE1 ACTIVE2 diff_OUTCOME seq;

run;

proc sort data=crossover; by seq;

proc univariate noprint data=crossover; by seq;

var diff_OUTCOME;

output out=xx1 mean=d var=v n=num std=s;

run;

data ttest;

do until (eof);

set xx1 end=eof;

if seq=1 then do; v1=v; n1=num; d1=d; sd1=s; end;

else if seq=2 then do; v2=v; n2=num; d2=d; sd2=s; end;

end;

dbar=(d1+d2)/2;

sdpool = sqrt( (v1*(n1-1) + v2*(n2-1)) / (n1+n2-2) );

stderr = sqrt( (sdpool**2)/4 * ((1/n1) + (1/n2)) );

t=dbar / stderr;

df=n1+n2-2;

p=(1-probt(abs(t),df))*2;

**--CALC 95% CI --**;

t_critical=tinv(0.975,df);

lower=(dbar - t_critical*stderr);

upper=(dbar + t_critical*stderr);

run;

title1 'Cross-over ttest to estimate treatment effect';

proc print data=ttest; var dbar sdpool stderr t df p lower upper; run;

*****************************************************************;

*** MIXED MODELS ***;

title1 'Model 1: (mixed model with an unstructured covariance matrix)';

title2 'with carryover term';

Proc mixed data = all asycorr asycov;

class patientid PERIOD2 ACTIVE CARRYOVER BASELINE;

model OUTCOME = PERIOD2 ACTIVE CARRYOVER BASELINE /solution ddfm = kr;

repeated/type = unr sub = patientid;

run;

title2 'NO carryover term';

Proc mixed data = all;

class patientid PERIOD2 ACTIVE BASELINE;

model OUTCOME = PERIOD2 ACTIVE BASELINE /solution ddfm = kr;

repeated/type = unr sub = patientid;

run;

title1 'Model 2: (mixed model with a compound symmetry covariance matrix)';

title2 'with carryover term';

Proc mixed data = all;

class patientid PERIOD2 ACTIVE CARRYOVER BASELINE;

model OUTCOME = PERIOD2 ACTIVE CARRYOVER BASELINE /solution ddfm = kr;

random int/type = un sub = patientid;

run;

title2 'NO carryover term';

Proc mixed data = all;

class patientid PERIOD2 ACTIVE BASELINE;

model OUTCOME = PERIOD2 ACTIVE BASELINE /solution ddfm = kr;

random int/type = un sub = patientid;

run;
